# Relationship of CD44^+^CD24^-/low^ breast cancer stem cells and axillary lymph node metastasis

**DOI:** 10.1186/1479-5876-10-S1-S6

**Published:** 2012-09-19

**Authors:** Wei Wei, Hui Hu, Haosheng Tan, Louis WC Chow, Adrian YS Yip, Wings TY Loo

**Affiliations:** 1 Department of Breast Surgery, Peking University Shenzhen Hospital, Shenzhen, Guangdong, China; 2 Organisation for Oncology and Translational Research and UNIMED Medical Institute, Hong Kong, China

## Abstract

**Background:**

Axillary node staging plays an important role in the prognostic evaluation and planning of adjuvant treatment. Breast cancer stem cells, identified on the basis of CD44^+^CD24^-/low^ expression, are associated with metastases and drug resistance. It is therefore important to investigate the proportion of CD44^+^CD24^-/low^ breast cancer stem cells for the diagnosis of metastases in axillary nodes.

**Methods:**

Thirty-two ipsilateral axillary lymph nodes were collected from patients diagnosed with invasive breast cancer. Each lymph node (LN) was divided into two equals – one was examined by H&E staining, while the other was made into a single cell suspension to study the content of CD44^+^CD24^-/low^ cells by flow cytometry (FCM). The relationship was investigated between the content of CD44^+^CD24^-/low^ cells and metastases in axillary nodes which were confirmed by histology. Associations were tested using the chi-square test (linear-by-linear association), and the significance level was set at a value of p < 0.05.

**Results:**

In the 32 axillary nodes, the level of CD44^+^CD24^-/low^ cells was determined to be between 0 and 18.4%: there was no presence of CD44+CD24-/low cells in 9 LNs, of which 2 had confirmed metastasis; there were less than 10% CD44+CD24-/low cells in 12 LNs, of which 6 had confirmed metastasis; and there were more than 10% CD44+CD24-/low cells in 11 LNs, of which 9 had confirmed metastasis. A higher percentage of detected CD44^+^CD24^-/low^ cells was significantly associated with more confirmed LN metastases (p = 0.009).

**Conclusions:**

CD44^+^CD24^-/low^ breast cancer stem cells might help clinicians to determine the presence of LN metastases. However, its prognostic value remains unclear, while histological diagnosis is still the gold standard.

## Background

Breast cancer stem cells (BCSCs) represent a minor subset of cells in a breast tumor that is deemed as the driver of tumor initiation, displaying resistance to drugs and developing metastatic disease [1]. BCSCs, identified on the basis of CD44^+^CD24^-/low^, Lin expression, could form tumors in nonobese, diabetic/severe combined immunodeficiency disease mice with as few as 200 of such cells [2]. The expression was strongly associated with poor clinical outcome and other prognostic indicators such as tumor histological grade, proliferation index, hormonal receptor expressions, and avian erythroblastosis oncogene B2 overexpression [3]. A high percentage of BCSCs enriched with CD44^+^CD24^-/low^ phenotype was found harbored in basal-like tumors [4] and the phenotype was observed in ‘triple-negative’ breast cancer that responded poorly to chemotherapy [5]. The combined expressions with CD44 associated with stem cell-like characteristics and CD24 related to differentiated epithelial features [6] as prognostic markers for breast cancer however remains controversial.

Positive locoregional LN has long been recognized as an indicator of regional metastases and, subsequently, a reservoir for further lymphatic and later visceral metastases [7-9]. It is well noted that tumor cells with CD44^+^CD24^-/low^ expression not only possess malignant behavior as other tumor cells, but also share with normal stem cells the capacity for self-renewal [3,10-12]. In a recent *in vitro* and *in vivo* study, a high proportion of BCSCs with CD44^+^CD24^-/low^ expression was presented in a model with high lymphatic metastatic ability [13]. The expression level of BCSCs might highlight the metastatic potential of the disease in the lymphatic system, the major route of breast cancer metastases.

Regional LN dissection has been regarded as a staging procedure rather than surgical treatment because it was not seen to benefit survival. [14,15]. The sentinel lymph node (SLN) biopsy has modified the need of axillary surgery in the treatment of breast cancer for patients; yet, it remains a staging procedure but reduces post-operative morbidity [16]. Accurate staging of ipsilateral LN involvement is, however, the best clinicopathologic prognostic indictor for oncologists to precisely evaluate and offer breast cancer patients the most suitable adjuvant therapy. Hematoxylin and eosin (H&E) staining of the surgical LN samples is still the current gold standard for diagnosing axillary LN metastasis. However, the process of creating paraffin-embedded slides involves many steps, leading to a prolonged wait for pathological results. In this study, we aimed to investigate the proportion of CD44^+^CD24^-/low^ BCSCs in axillary LN and examined the relationship with H&E staining results.

## Methods

### Patients

The study was approved by the local ethics committee of the Peking University Shenzhen Hospital. Between April 2008 and April 2009, a total of 32 cases were prospectively sampled for ipsilateral axillary LNs at the Department of Breast Surgery of Peking University Shenzhen Hospital. All patients were diagnosed with invasive breast cancer, confirmed by histological assessment and were scheduled for surgery. Women who previously received chemotherapy or endocrine therapy for breast cancer were excluded from the trial. The median age of female patients enrolled into the trial was 42 years (range, 21-69 years).

### Antibodies and materials

Mouse-anti-human IgG1-FITC (No. : 11-4714), IgG2b-PE-CY5 (No. : 15-4031), CD24-FITC (No. : eBioSN3), CD44-PE-CY5 (No. : IM7), and CD326-PE (No. : 1B7) were obtained from eBioscience, USA; collagenase III, fetal bovine serum, and OptilyseC hemolytic agents were obtained from GIBICO, USA. Flow cytometry: Epics XL, Beckman Coulter were used.

### Preparation of single cell suspension

The entire ipsilateral axillary LNs – swollen and hard in texture – were obtained from patients after surgical resection within one hour. Each node was divided into two equal parts. One was taken for histopathological examination employing H&E staining, and the other was prepared for a single cell suspension. Adipose tissue of the LN was removed using ophthalmic scissors, and the specimen was washed repeatedly using phosphate buffer solution (PBS) until the blood was totally removed. The washed specimen was then placed in a Petri dish which was put on ice, fixed by ophthalmic scissors, repeatedly pricked using No. 9 syringe needles and rinsed with small amounts of PBS until the sample turned filamentous. After, the specimen was repeatedly stamped using two tissue forceps and rinsed with small amounts of PBS. The washing liquid was collected and centrifuged at 1500 r/min for five minutes. The supernatant was removed and one ml of PBS was added for re-hanging. Afterward, 3 - 5ml of 0.1% collagenase III was added, and the sample was placed into a 37 °C water bath for 10-30 minutes, during which the sample was pipetted once every 5 minutes using a 10ml pipette. Fetal bovine serum (FBS) was then added to a final concentration of 10% and centrifuged at 1500 r/min for 5 minutes. Supernatant was removed and 2ml PBS was added for re-suspension, and then filtered through 300-mesh stainless steel sieve. The filtered fluid was added with 0.75ml OptilyseC and left to stand for 15 minutes. Supernatant was removed after the fluid was added and centrifuged at 1500 r/min for 5 minutes; afterward, PBS was added for cleaning purposes. Finally, 100ul PBS was added for re-suspension.

### Cell mark and flow cytometry test

Antibodies IgG1-FITC 10ul, CD326-PE 10ul, IgG2b-PE-CY5 10ul and cell suspension 50ul were added into control EP tubes, while antibodies CD24-FITC 10ul, CD326-PE 10ul, CD44-PE-CY5 10ul and cell suspension 50ul were added into target EP tubes. The tubes were incubated at room temperature for 15 minutes in the shade. Afterward, the tubes were washed with PBS and 500ul PBS was added for re-suspension. The voltage of the control was reset with CD326-PE/SSC to detect the proportion of CD44^+^CD24^-/low^ cells in CD326-positive cell populations.

### Statistical analysis

Chi-square test (linear-by-linear association) was used to examine the relationship between CD44^+^CD24^-/low^ cell contents and the axillary LN metastasis using SPSS for windows release 13.0 (SPSS Inc., USA). The significance level was set for p-value less than 0.05.

## Results

**Figure 1:**
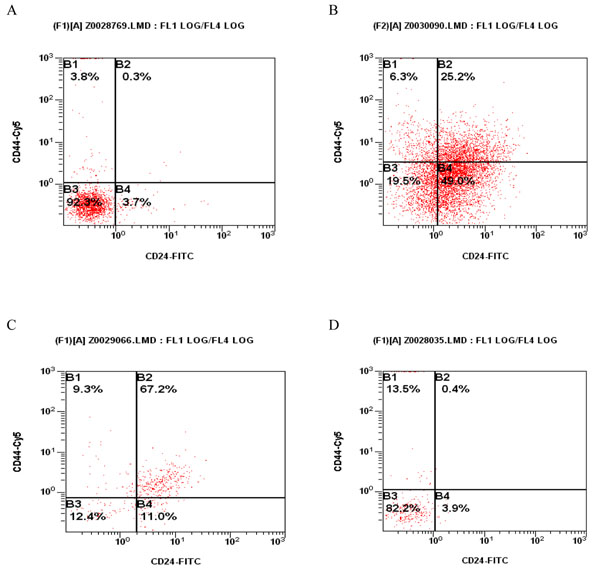
**Typical content of CD44^+^CD24^-/low^ cells in axillary LNs** A and B: With micro-metastasis; C and D: Absent of micro-metastasis

The mechanical-enzymatic method was employed to make 32 fresh LN tissue samples into a single cell suspension. The CD44^+^CD24^-/low^ cell content was detected using flow cytometry and compared with H&E staining of paraffin section. In 9 LNs in which CD44^+^CD24^-/low^ cell content was 0%, 2 were confirmed with metastasis. In 12 LNs where CD44^+^CD24^-/low^ cell content was between 0% and 10/%, 6 were confirmed with metastasis. In 11 LNs in which CD44^+^CD24^-/low^ cell content was between 10% and 18.4%, 9 were confirmed with metastasis (Table 1 and figure 1). It can therefore be concluded that a higher percentage of CD44^+^CD24^-/low^ cells detected in axillary LN was significantly associated with a higher chance of LN metastasis (p = 0.009).

**Table 1:** Comparison of CD44^+^CD24^-/low^ cell content and axillary LNs metastases confirmed by H&E staining from 32 cases

CD44^+^CD24^-/low^ cell content (%)	Number of axillary lymph nodes	Metastases confirmed in paraffin sections	Percentage of positive axillary lymph node (%)
0	9	2	22.2

0ï½ž10	12	6	50.0

10ï½ž18.4	11	9	81.8

## Discussion

Cancer stem cell is an ancient but everlasting topic, where hypotheses about cancer stem cells existed as early as 100 years ago [17-19] and the discovery of leukemia stem cells has suggested distinct cell subpopulations [20]. In 1997, Bonnet *et al.* isolated CD34CD38 cells in human acute myeloid leukemia [20]. In recent years, scientists were able to isolate stem-like cells (tumor initiating cells) from solid tumors. These findings not only laid the theoretical foundation of cancer stem cells, but also shed light for the treatment of malignant tumor. For a long time, treatment strategies for malignancy were mainly aimed at tumor cells in the proliferation and mitotic phases. This leads cancer cells into interphase, the main cause of cancer recurrence and metastasis. Cancer stem cell theory has changed the traditional treatment strategy for patients and will one day completely cure malignant tumors [21].

As researchers are able to isolate ESA^+^ CD44^+^CD24^-/low^ LIN^-^ cells from breast cancer tissues [3], more and more studies on BCSCs have appeared, particularly on CD44^+^CD24^-/low^ cell’s clinical correlation. However, there is little research about its clinical value. The mechanical-enzymatic method was employed to transform 33 fresh LN tissue samples into a single cell suspension, the CD44^+^CD24^-/low^ cell content was then detected using flow cytometry and compared with H&E stained paraffin section to explore the relationship between CD44^+^CD24^-/low^ cell content and LN metastasis.

### Process of breast cancer metastasis to axillary LNs

Invasive breast cancer cells first invade lymphatic vessels and then travel to local LNs through the lymphatic flow system. The first involved nodes are one or a few pectoral LNs, known as SLN. In the LNs, cancer cells first cluster in marginal sinus, then accumulate in the entire LN. This causes LNs to swell and harden, appearing gray in sections. Sometimes, tumor tissues invade capsules and cause several LNs to merge together. After regional LN metastasize, tumor cells continue to transfer to the next nodes, eventually entering the blood vessel through the thoracic duct and secondary metastasis might occur [22].

Contamination of blood cells, cell debris and other particles in a single cell suspension may cause nonspecific bindings and influence the result. They may be wrongly labeled by fluorescent monoclonal antibodies, resulting in non-specific binding and thus inducing experimental errors. ESA (CD326), also known as epithelial-specific antigen (EpCAM), is cell surface glycoprotein of 40Kda in weight. It’s a broad spectrum marker of epithelial cells [23]. In our study, we employed CD326 (ESA) / SS instead of FS / SS as a means of determining ESA-positive cells and to decrease the influence of cell debris and blood cells. After the detection scheme was established, we tested the scheme using breast cancer cell lines and determined the preliminary parameters to minimize experimental errors. In our study, the specificity of epithelial cell marker CD326 was about 80%. Normal lymphoid tissue may still contain a small amount of other endothelial cells after processing; therefore, false positive results may still arise during the measurement of CD44^+^CD24^-/low^ cell contents when representing the proportion of BCSCs in axillary LN metastasis. A low proportion of breast cancer cells present in LNs may also result in the absence of specific binding with CD326 monoclonal antibody and in turn, the accuracy of detecting CD44^+^CD24^-/low^ cell contents in SLN was reduced.

### Relationship of CD44+CD24^-/low^ cell content and axillary LN metastasis

In 2005, Abraham BK *et al.* confirmed the proportion of CD44^+^CD24^-/low^ cells was associated with distant metastasis using immunohistochemical staining [24]. In 2008, Ling LJ *et al.* confirmed that a tumor having a higher proportion of CD44^+^CD24^-/low^ cells is more prone to bone metastasis using animal models [25]. It was therefore postulated that the content of CD44^+^CD24^-/low^ cells or, in other words, the proportion of BCSCs might be related to tumor aggressiveness and distant metastasis, but the relationship with axillary LN status was not well established. It is possibly because lymphatic circulation is slower and shorter than blood circulation and, in addition to invasive stem cells, non-stem cells can also metastasize to ipsilateral axillary LNs. Compared to stem cells, non-stem cells have a smaller chance of metastasizing according to their weaker degree of aggressiveness. It was speculated that distant metastasis of breast cancer is mainly caused by stem cells.

### Diagnosis value of CD44+CD24^-/low^ cells in axillary LN metastases

Currently, common LN micro-metastasis markers of breast cancer are human mammaglobin (hMAM) and adhesion protein 1, where hMAM is known to have high specificity and sensitivity. Marchetti A *et al.*[26] detected LNs using the real-time polymerase chain reaction (RT-PCR) laboratory technique, and only hMAM and CEA mRNA were not detected in LNs of healthy individuals, therefore concluding that the above two markers have high specificity. Mitas M *et al*. [27] demonstrated that hMAM was over-expressed in 16/17 pathology-negative LNs, suggesting that it could be used as marker of breast cancer micrometastases in LNs. Mucin, a Mucin 1 mRNA encoding product, is a membrane glycoprotein with transmembrane domain. Mucin 1 has become an indicator of LN micrometastasis in breast cancer patients. Hao XB *et al. *[28] found that mucin 1 mRNA was expressed in the LNs of all 86 breast cancer patients and RT-PCR did not show mucin 1 expression in LNs with benign disease. However, results are inconsistent. Marchetti A *et al. *[26] detected the expression of mucin 1 mRNA in healthy LNs, but with low specificity. These two indicators for LN metastasis of breast cancer markers may only relate to the distribution of organs, while CD44^+^CD24^-/low^ cells not only relate to the distribution of organs, but also to their biological characteristics (invasiveness). Compared with hMAM and mucin 1, CD44^+^CD24^-/low^-expressing BCSCs may have higher false negative and lower false positive results.

## Conclusions

In conclusion, this study suggests that in using CD44^+^CD24^-/low^ cells to estimate the metastasis of axillary LNs, a higher content corresponds to lower false-positive results. As CD44^+^CD24^-/low^ double-labeling LN metastases in cell distribution and function, it may have lower false positive rates compared to existing markers. Further investigation on the relationship between the CD44^+^CD24^-/low^ expression of tumor cells and LN metastases is warranted. The detection of BCSCs in LNs may be useful in predicting micrometastases, especially in sentinel LNs, as a routine supplementary pathological reference.

## Competing interests

The authors declare that they have no competing interests.

## Authors' contributions

WW conducted the study. AY participated in manuscript writing. WL performed data analysis and participated in writing. HH conducted the study. HT conducted the study. LC performed data analysis.
